# Clustering Scatter Plots Using Data Depth Measures

**DOI:** 10.4172/2155-6180.S5-001

**Published:** 2011-12-25

**Authors:** Zhanpan Zhang, Xinping Cui, Daniel R Jeske, Xiaoxiao Li, Jonathan Braun, James Borneman

**Affiliations:** 1 Department of Statistics, University of California, Riverside, CA, USA; 2 Department of Molecular and Medical Pharmacology, University of California, Los Angeles, CA, USA; 3 Department of Pathology and Laboratory Medicine, University of California, Los Angeles, CA, USA; 4 Department of Plant Pathology and Microbiology, University of California, Riverside, CA, USA

**Keywords:** Clustering, Scatter Plot, Data Depth, Quality Index, Visualization

## Abstract

Clustering is rapidly becoming a powerful data mining technique, and has been broadly applied to many domains such as bioinformatics and text mining. However, the existing methods can only deal with a data matrix of scalars. In this paper, we introduce a hierarchical clustering procedure that can handle a data matrix of scatter plots. To more accurately reflect the nature of data, we introduce a dissimilarity statistic based on “data depth” to measure the discrepancy between two bivariate distributions without oversimplifying the nature of the underlying pattern. We then combine hypothesis testing with hierarchical clustering to simultaneously cluster the rows and columns of the data matrix of scatter plots. We also propose novel painting metrics and construct heat maps to allow visualization of the clusters. We demonstrate the utility and power of our new clustering method through simulation studies and application to a microbe-host-interaction study.

## Introduction

Clustering is rapidly becoming a powerful data mining technique, and has been broadly applied to many domains such as bioinformatics [1,2] and text mining [3]. Usually the data are arranged in a data matrix where each row corresponds to an object and each column to a variable on which objects are characterized. Each element of this matrix is a real number, representing the measurement of an object on a specific variable. To avoid confusion, we call this matrix “the data matrix of scalars”.

Two one-dimensional clustering methods are commonly used: Hierarchical clustering builds a hierarchy of clusters based on the dissimilarity measures among objects whose results can be graphically presented in a tree structure, called dendrogram; Partitioning clustering, such as k-means, divides the objects into a pre-specified number of clusters in which each object belongs to the cluster with the nearest mean. One may see [4,5] for a survey.

Co-clustering, also called biclustering, bivariate clustering, or two-mode clustering, is to simultaneously cluster rows and columns. Unlike the one-dimensional clustering methods that seek to identify similar rows or columns independently, co-clustering seeks to identify “blocks” (or “co-clusters”) of rows and columns that show highly inter-related coherence. For example, in gene expression analysis, co-clustering can be used to solve the dual problem of identifying a set of genes and conditions simultaneously involved in a metabolic process, a problem that traditional one-dimensional clustering methods can not handle. Reference [6–9] showed a detailed review.

However, when each cell of the data matrix is not represented by a single numerical value and instead contains a scatter plot, all the existing clustering methods are not applicable any more. One may think of incorporating the current clustering methods by using a single measure, say Pearson correlation coefficients, to analyze the associations between row variables and column variables, which then reduces the data matrix of scatter plots to the data matrix of scalars. But the choice of Pearson correlation coefficients is not always sufficient since it is only a measure of linear association and it is very sensitive to outliers. Therefore, distance measures among objects based on such coefficients will hinder the power of discovering clusters of scatter plots with nonlinear patterns and/or clusters with outliers.

In this paper we introduce a hierarchical clustering procedure that can handle a data matrix of scatter plots. In Section 2, to more accurately reflect the nature of data, we introduce a dissimilarity statistic based on “data depth” to measure the discrepancy between two bivariate distributions without oversimplifying the nature of the underlying pattern. We then combine hypothesis testing with hierarchical clustering to simultaneously cluster the rows and columns of the data matrix of scatter plots. We also propose novel painting metrics and construct heat maps to allow visualization of the clusters. In Section 3 and 4, we demonstrate the utility and power of our new clustering method through simulation studies and application to a microbe-host-interaction study.

## Methodology

### Clustering procedure

Consider a set of row variables {*X_1_,X_2_,..,V_M_*} and a set of column variables {*Y_1_,Y_2_,..,Y_N_*}. For each pair of row and column, a number of observations are taken that can be drawn as a scatter plot in the Cartesian plane. Our goal is to cluster both rows and columns based on these *M×N* scatter plots.

**Figure 1:**
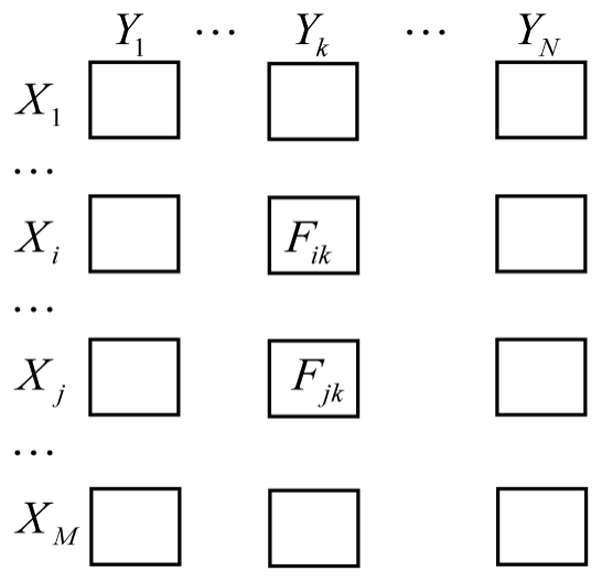
Data structure: a data matrix of scatter plots.

To obtain the distance matrix for performing the hierarchical clustering of rows, we have to calculate the distance between any two rows. Consider the *i^th^* row and the *j^th^* row, we would like to measure how similar these two rows are to each other based on comparing the corresponding N pairs of scatter plots. For each column, say the *k^th^* column, the pair of scatter plots can be thought of as the samples taken from two independent bivariate distributions *F_ik_* and *F_jk_* respectively, as shown in (Figure 1) in which each square contains a scatter plot. As a result, the problem of comparing the pair of scatter plots can be formulated as testing the following hypotheses:

(1) $${\text{H}}_{0}:F_{tk}=F_{jk}\;vs.H_{a}:F_{ik}\neq F_{jk}.$$

Denote by (*p_value_*)*_ijk_* the p-value for testing the above hypotheses. The smaller the p-value, the less similar the pair of scatter plots to each other. By testing the same kind of hypotheses for all the N columns, we define the dissimilarity (distance) between the ith row and the jth row as

(2) $${\mathrm{dist}}_{ij}=\sum_{k=1}^{N}\left(1-{\left(\mathrm{pvalue}\right)}_{\mathrm{ijk}}\right).$$

Then the distance matrix for rows {*dist_ij_*} (*i, j = 1,2,..,M, and i* ≠ *j*) is inputted to the regular hierarchical clustering algorithm, which initially regards each row as an individual cluster, and at each step, merges the closest pair of clusters until all the rows are merged into one cluster. In doing this, hierarchical clustering creates a hierarchy of row clusters that can be represented in a tree structure called dendrogram.

The same clustering procedure can be applied to columns as well. Therefore, the rows and the columns in the original data matrix of scatter plots (Figure 1) are reordered according to the row dendrogram and the column dendrogram, respectively, which produces a new data matrix of scatter plots that acts as the output of our proposed clustering procedure.

### Hypotheses testing

Liu RY, Singh K [10] proposed a multivariate rank sum test for the hypotheses H_0_: *F_ik_ = F_jk_ vs. Ha: F_ik_*≠*F_jk_* where *F_ik_* and *F_jk_* are the distribution functions of two independent populations. Specifically, the test statistic is based on a quality index that measures the overall “outlyingness” of population *F_jk_* relative to population *F_ik_*,

(3) $$Q(F_{ik},F_{jk})=P\left(D(F_{ik};\vec{U})\leq D(F_{ik};\vec{V})|\vec{U}\sim F_{ik},\vec{V}\sim F_{jk}\right),$$

where *D*(*F_ik_*;·) is an affine-invariant data depth function with respect to *F_ik_* that could be Mahalanobis depth, Tukey (Halfspace) depth, and Simplicial depth, etc. (Refer to Section 6.1 for details)

Given two samples {*U⃗*_1_*,* ···, *U⃗_S_*} from *F_ik_* and {*V⃗*_1_*,* ···, *V⃗_T_*} from *F_jk_*, Q(*F_ik_*,, *F_jk_*) can be estimated as

(4) $$Q(F_{ik}^{S},F_{jk}^{T})=\frac{1}{T}\sum_{t=1}^{T}R(F_{ik}^{S};{\vec{V}}_{t}),$$

where $F_{ik}^{S}$ and $F_{jk}^{T}$ are the empirical distributions, $R(F_{ik}^{S};{\vec{V}}_{t})$ is *U⃗_s_*’s the proportion of with $D(F_{ik}^{S};{\vec{U}}_{s})\leq D(F_{ik}^{S};{\vec{V}}_{t})$, and $D(F_{ik}^{S};\cdot )$ is the empirical data depth with respect to. From [10,11], we have

(5) $$Q(F_{ik}^{S},F_{jk}^{T})-1/2\sim AN(0,(1/S+1/T)/12)$$

under H_0_: *F_ik_* = *F_jk_* for many commonly used data depth functions (under general regularity conditions).

Notice that the overall “outlyingness” of *F_ik_* relative to *F_jk_* can be also measured by a quality index

(6) $$Q(F_{jk},F_{ik})=P\left(D(F_{jk};\vec{V})\leq D(F_{jk};\vec{U})|\vec{V}\sim F_{jk},\vec{U}\sim F_{ik}\right),$$

where *D*(*F_jk_*;·) is an affine-invariant data depth function with respect to *F_jk_*. Likewise, Q(*F_jk_*,, *F_ik_*) may be estimated as

$$Q(F_{jk}^{T},F_{ik}^{S})=\frac{1}{S}\sum_{s=1}^{S}R(F_{jk}^{T};{\vec{U}}_{s}),$$

where $R(F_{jk}^{T};{\vec{U}}_{s})$ is the proportion of *V⃗_t_*’s with $D(F_{jk}^{T};{\vec{V}}_{t})\leq D(F_{jk}^{T};{\vec{U}}_{s})$, and $D(F_{jk}^{T};\cdot )$ is the empirical data depth with respect to $F_{jk}^{T}$.

It can be shown that *Q*(*F_jk_*, *F_ik_*) is not directly related to *Q*(*F_ik_*,, *F_ik_*) (Refer to Section 6.2 for more explanation). However intuitively, we would like to have a unique parameter to measure the difference between two distributions, either comparing *F_ik_* to *F_jk_*, or *F_jk_* to *F_ik_*. Under H_0_: *F_ik_* = *F_jk_*, Q(*F_ik_*,, *F_ik_*) = Q(*F_jk_*,, *F_ik_*) = ½. With the location shift and/or scale change between *F_ik_* and *F_jk_*, either Q(*F_ik_*,, *F_ik_*) or Q(*F_jk_*,, *F_ik_*), or both, would deviate from 1/2 relatively significantly. Therefore, to avoid having one distribution as the reference distribution, we propose a new quality index, called TS, to measure the overall “difference” between *F_ik_* and *F_jk_*,

(8) $$TS=\left\{ \begin{array}{c} Q(F_{ik},F_{jk}),\text{if}\;|Q(F_{ik},F_{jk})-1/2|>|Q(F_{jk},F_{ik})-1/2|;\\ Q(F_{jk},F_{ik}),\text{if}\;|Q(F_{ik},F_{jk})-1/2|<|Q(F_{jk},F_{ik})-1/2|. \end{array}\right.$$

The test statistic for testing H_0_: *F_ik_* = *F_jk_* vs. Ha: *F_ik_*≠*F_jk_* is the estimate of *TS*,

(9) $$\hat{TS}=\left\{ \begin{array}{c} Q(F_{ik}^{S},F_{jk}^{T}),\text{if}\;|Q(F_{ik}^{S},F_{jk}^{T})-1/2|>|Q(F_{jk}^{T},F_{ik}^{s})-1/2|;\\ Q(F_{jk}^{T},F_{ik}^{S}),\text{if}\;|Q(F_{ik}^{S},F_{jk}^{T})-1/2|<|Q(F_{jk}^{T},F_{ik}^{S})-1/2|. \end{array}\right.$$

Then (*pvalue*)*_ijk_* is calculated by the following permutation test procedure:

1. Pool two samples {*U⃗*_1_, ···;, *U⃗_S_*} and {*V⃗*_1_, ···, *V⃗_T_*}.
2. Take a sample of size *S* without replacement { ${\vec{U}}_{1}^{*},\cdots ,{\vec{U}}_{S}^{*}$} from the pooled sample, and the remaining is { ${\vec{V}}_{1}^{*},\cdots ,{\vec{V}}_{T}^{*}$}, which are called two permutation samples.
3. Estimate *Q*(*F_ik_*, *F_ik_*) and *Q*(*F_jk_*, *F_ik_*) by $Q^{*}(F_{ik}^{S},F_{jk}^{T})$ and $Q^{*}(F_{jk}^{T},F_{ik}^{S})$, respectively, based on the permutation samples obtained in Step 2.
4. Set ${\hat{TS}}^{*}$ to be equal to $Q^{*}(F_{ik}^{S},F_{jk}^{T})$ if $|Q^{*}(F_{ik}^{S},F_{jk}^{T})-1/2|>|Q^{*}(F_{jk}^{T},F_{ik}^{S})-1/2|$; and equal to $Q^{*}(F_{jk}^{T},F_{ik}^{S})$ otherwise.
5. Repeat the above steps (Step 2 – Step 4) *B* times to yield *B* values of ${\hat{TS}}^{*}$, denoted by ${\hat{TS}}_{b}^{*}(b=1,2,\cdots B)$, whose distribution estimates the sampling distribution of the test statistic $\hat{TS}$ under H_0_: *F_ik_* = *F_jk_*.
6. Let *p_lower_* be the proportion of ’s with, and pupper the proportion of ’s with. Hence (*p_value_*)*_ijk_* = 2 × min(*p_lower_*, *p_upper_*).

### Data visualization

Data visualization is an important aspect in the clustering technique. In the traditional hierarchical clustering application, where cells in a data matrix are scalars, the original data can be rearranged according to the dissimilarity scores between rows (or columns). The smaller the dissimilarity score between two rows (or columns), the closer the two rows (or columns). A graphical representation of the rearranged data matrix, called a heat map, can be created where the cells are color coded based on their scalar values. Obviously, we would expect cells in close proximity to each other to have a similar color.

However, it is not straightforward to apply the above painting strategy to a data matrix of scatter plots since scatter plots can not be distinguished from each other only by a single color painting system. In the following we introduce three painting metrics and demonstrate how to use these metrics to graphically represent the clusters of scatter plots so that similar scatter plots share the similar color painting whereas different scatter plots correspond to different color paintings.

**Figure 2:**
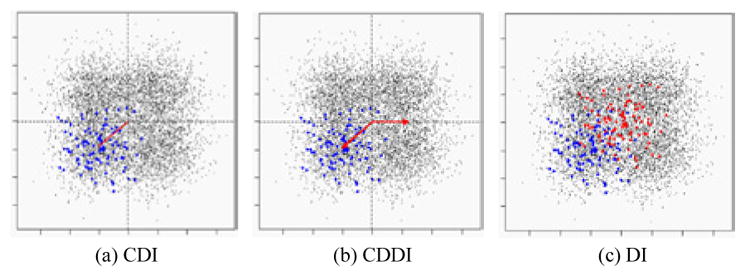
Three painting metrics.

**Figure 3:**
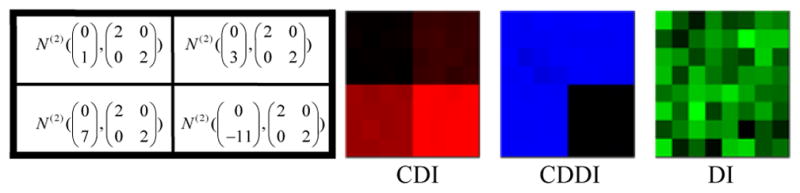
Painting Example 1: the bivariate normal distributions differ by location only and are asymmetric about the origin, therefore only CDI can reveal clusters of scatter plots.

**Figure 4:**
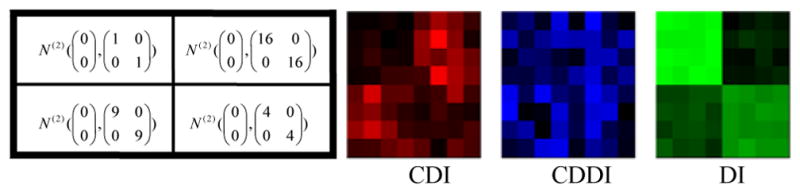
Painting Example 2: the bivariate normal distributions differ by location only and are symmetric about the origin, therefore only CDDI can reveal clusters of scatter plots.

**Figure 5:**
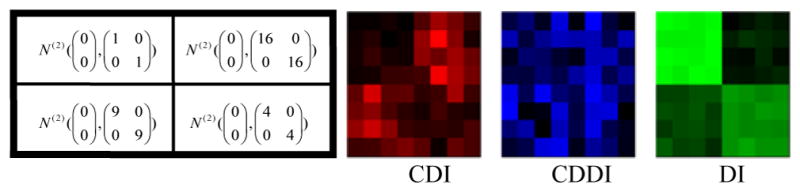
Painting Example 3: the bivariate normal distributions differ by scale only, therefore only DI can reveal clusters of scatter plots.

**Figure 6:**
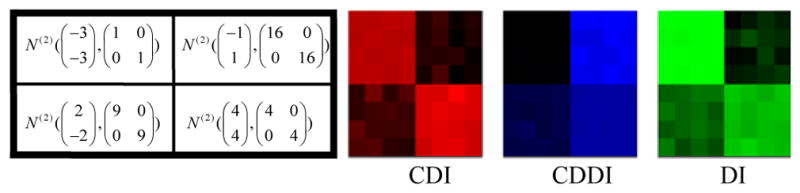
Painting Example 4: the bivariate normal distributions differ by both location and scale and asymmetric about the origin, therefore CDI, CDDI and DI all reveal clusters of scatter plots.

1. **Center Deviation Index (CDI):** All the *M×N* scatter plots are pooled as a single scatter plot that is thought of as a sample from the bivariate distribution *F_pool_*. For any scatter plot that is a sample from the bivariate distribution, we define its center as the point that maximizes the empirical data depth for. Then the CDI for a scatter plot is the distance between its center and the center of the pooled scatter plot. For example, in (Figure 2a), the length of red segment is the CDI measuring the deviation of the scatter plot consisting of blue points from the pooled scatter plot consisting of black points.
2. **Center Deviation Direction Index (CDDI):** By taking the center of the pooled scatter plot as the origin of a new Cartesian coordinate system, the CDDI for a scatter plot is the magnitude of the angle formed by the vector from the origin to its center and the positive *x-axis*, which ranges from −π to π. The CDDI depicts the relative location of a scatter plot with respect to the pooled scatter plot, and then the relative locations among the scatter plots. For example, in (Figure 2b), the CDDI for the blue scatter plot is the degree of the angle formed by two red vectors.
3. **Dispersion Index (DI):** Consider a scatter plot that is regarded as a sample from the bivariate distribution G. We move this scatter plot such that its center and the center of the pooled scatter plot overlap, which produces a shifted scatter plot that is regarded as a sample from a new bivariate distribution G′. The DI for the original scatter plot is the estimation of the quality index *Q(F_pool_, G′)*, which accounts for the difference between the original scatter plot and the pooled scatter plot excluding the effect due to the location shift. For example, in (Figure 2c), the DI for the blue scatter plot is the estimated quality index of the red scatter plot (obtained from moving the blue scatter plot) with respect to the pooled scatter plot.
  To better understand the utility of the above three painting metrics, we present a number of painting examples. In each example, an matrix of scatter plots (each scatter plot contains 100 data points) was generated with the top left 4×4, top right 4×4, bottom left 4×4 and bottom right 4×4 scatter plots following the four different distributions specified in the left panel of (Figure 3,4,5,6). We then obtained 8×8 matrices of CDI, CDDI and DI, based upon which three heat maps can be easily generated as shown in the right panel of (Figure 3,4,5,6), where the “red” heat map is based on CDI, the “blue” heat map on CDDI, the “green” heat map on DI, and the “black” color stands for the minimum index value in all the three heat maps. Note that for simplicity, we used Mahalanobis depth in all the examples discussed here.

## Simulation study

To investigate the power of our proposed clustering method, we performed a class of simulation studies. The basic procedure is as follows:

1. Specify a “checkerboard” data pattern with a set of row clusters and column clusters in which each block shares the same bivariate distribution within itself;
2. Generate random samples based on the given bivariate distributions, and create a data matrix of scatter plots;
3. Apply our proposed clustering method to this data matrix of scatter plots, and check whether the original data pattern can be retrieved or not. That is, we check whether rows within the same block are still close to each other compared to other rows in the row dendrogram, and columns as well; or equivalently, whether there exists a cutting of row dendrogram such that the generated branch set are exactly same as the original set of row clusters, and columns as well;
4. Repeat Step 2 – Step 3 a number of times, and record the success rate, the proportion of times that we succeed in retrieving the original data pattern, which acts as the power measurement for our proposed clustering method.

**Figure 7:**
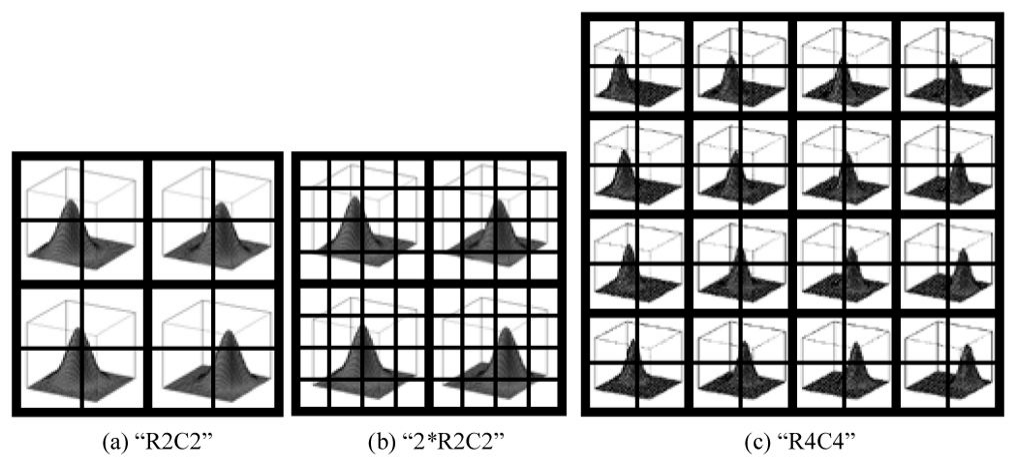
Three data pattern settings: (a) “R2C2”: there are 2×2 blocks (2 row clusters and 2 column clusters), each of which contains 2×2 cells, thus the data size is 4×4. (b) “2*R2C2”: the block size is doubled in the “R2C2” setting, thus the data size is 8×8. (c) “R4C4”: there are 4×4 blocks (4 row clusters and 4 column clusters), each of which contains 2×2 cells, thus the data size is 8×8.

Intuitively, the total number of rows and columns (the size of the data matrix of scatter plots, or the data size), the number of rows and columns within each block (the block size), and the number of blocks would affect the success rate. Therefore, we considered three data pattern settings shown in (Figure 7).

**Figure 8:**
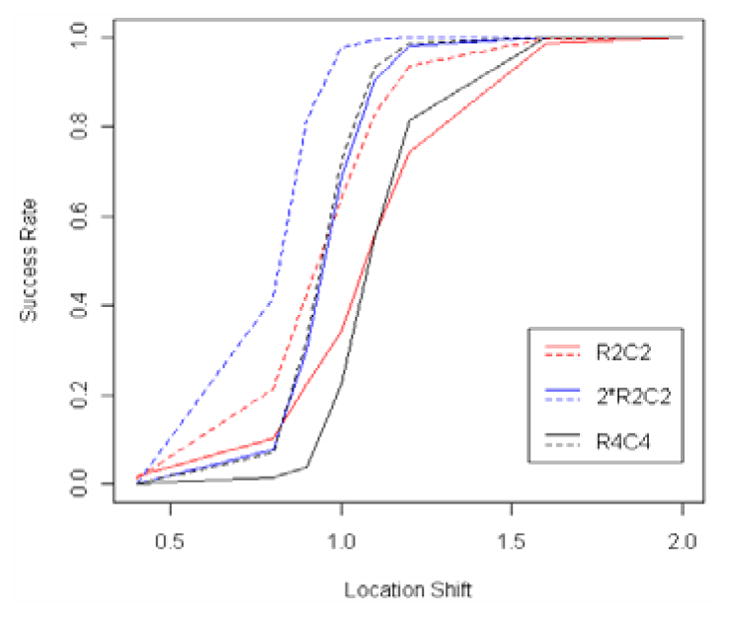
Success rate versus location shift.

For each setting, we specified a class of bivariate normal distributions for blocks, which only differ in location. Specifically, the *x*-coordinate of the mean increases equidistantly along the row direction ranging from 0 with the y -coordinate of the mean remaining same; whereas the *y*-coordinate of the mean increases equidistantly along the column direction ranging from 0 with the *x*-coordinate of the mean remaining same. For example, with a location shift of 1, the mean of the top left bivariate normal distribution in “R2C2” and “2*R2C2” is $\left(\begin{array}{c} 0\\ 0 \end{array}\right)$, the top right *ac*, the bottom left $\left(\begin{array}{c} 0\\ 1 \end{array}\right)$, and the bottom right $\left(\begin{array}{c} 1\\ 1 \end{array}\right)$. Furthermore, 50 data points were generated for each scatter plot, Mahalanobis depth was adopted, 500 resampling times were taken for the permutation test, and the “average” linkage method was chosen for the hierarchical clustering procedure. We performed 500 simulations for each setting. The relationship between the success rate and the location shift is summarized in (Figure 8), where the solid lines stand for the variance-covariance matrix $\left(\begin{array}{cc} 2 & 0\\ 0 & 2 \end{array}\right)$ specified for the bivariate normal distributions, and the dashed lines for $\left(\begin{array}{cc} 2 & 1\\ 1 & 2 \end{array}\right)$ with the correlation coefficient ρ = 0.5.

From (Figure 8), we may observe the following:

1. By comparing the solid line with the dashed line for each setting, the correlation in the bivariate normal distribution improves the success rate.
2. By comparing “R2C2” with “2*R2C2” both having a fixed number of blocks, with a relatively large location shift, the larger the block size, the higher the success rate; with a relatively small location shift, the smaller the block size, the higher the success rate. That is, more scatter plots with larger distance between blocks improves the chance of capturing the pattern. However, more scatter plots with smaller distance between blocks introduces a higher chance for noise in the clustering.
3. By comparing “2*R2C2” with “R4C4” both having a fixed data size, the smaller the number of blocks, the higher the success rate, which means it is harder to do a more delicate job (more row clusters and column clusters).
4. By comparing “R2C2” with “R4C4” both having a fixed block size, with a relatively small location shift, the smaller the number of blocks, the higher the success rate; with a relatively large location shift, the larger the number of blocks, the higher the success rate. The reason is similar to what we previously discussed in the comparison of “R2C2” with “2*R2C2”.

## Application

Our new clustering method should have utility for a variety of biological applications, one of which will be examining host-microbe interactions. For example, consider the case of inflammatory bowel disease (IBD). IBD is a disease that is likely caused by several factors, including genetics, lifestyle and intestinal bacteria. To identify putatively important host-microbe interactions, we recently examined the amounts of bacteria and proteins in mucosal luminal interface samples from IBD and healthy subjects [12].

Two datasets were generated from the experiment. “Microbe” data were arranged as a data matrix with 81 rows (3 rows containing missing values are excluded) standing for samples, 15 columns for microbes, and each cell being a single numerical value recording the level of a microbe in a sample. “Protein” data were also arranged as a data matrix with 81 rows standing for the same set of samples, 440 columns for proteins, and each cell being a single numerical value recording the level of a protein in a sample. To identify associations between levels of the microbes and proteins, we combined the above two data matrices of scalars by treating each pair of rows (one from “Microbe” data, the other from “Protein” data) as bivariate data with the y-axis being microbe level and the y-axis being protein level, which results in a data matrix of scatter plots as shown in (Figure 1) where *M=440*, *N=15*, and each scatter plot contains 81 data points.

Regarding the scatter plots as samples taken from the corresponding independent bivariate distributions, we applied our proposed clustering method to these 440×15 scatter plots, and cluster both proteins (rows) and microbes (columns). Specifically, we used Mahalanobis depth as the data depth measure, B=500 resampling times for the permutation test, and the “average” linkage method to perform the hierarchical clustering.

**Figure 9:**
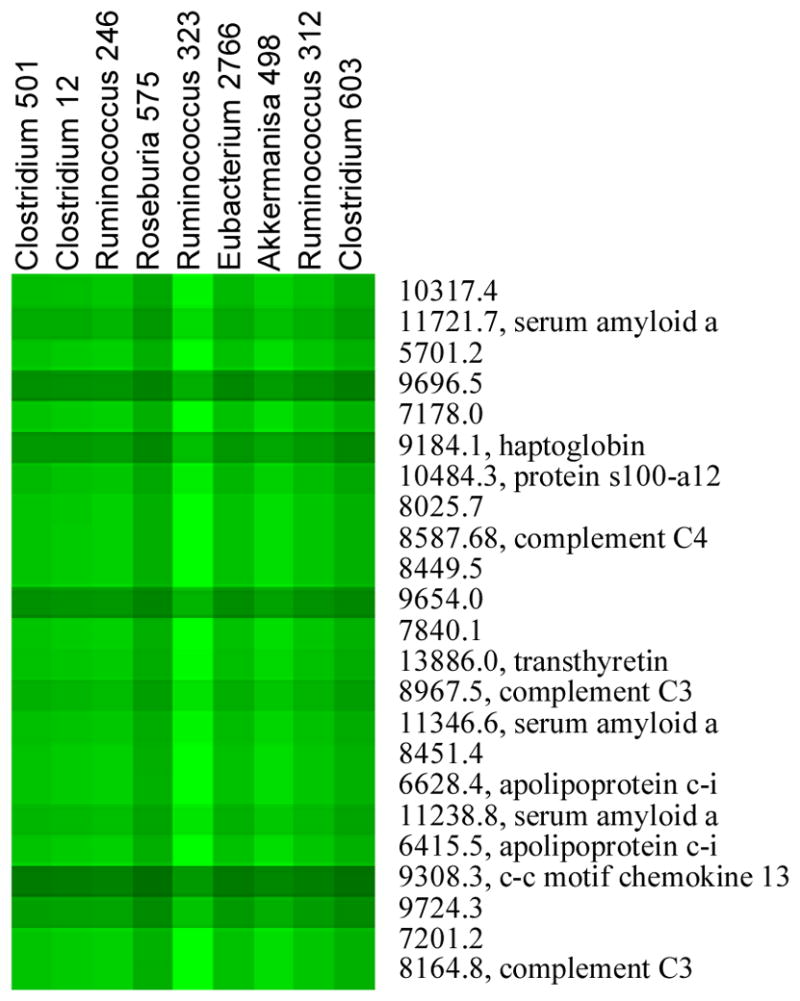
Heat map with the DI painting metric.

We then cut the “Protein” dendrogram at the height of 6, which generates 80 protein branches/clusters. The proteins within the same branch are more similar to each other, or show more similar microbe-protein patterns, than those in other branches. From the 80 protein clusters, we only selected those containing at least 20 proteins, which leads to 5 protein clusters. We also generated 4 microbe clusters by cutting the “Microbe” dendrogram at the height of 430, and selected those containing at least 5 microbes. One pair of the selected protein cluster and microbe cluster is depicted in (Figure 9), where the heat map with the DI painting metric is shown. The promise of these results is demonstrated by the fact that most of the identified proteins have been previously associated with IBD as in [13–18].

Examining such relationships will have utility for several purposes. First, by clustering relationships of various host and microbial variables, one can identify groups of relationships that have similar and/or dissimilar associations by visually examining the heat maps. Large assemblages of individual relationships with similar associations may point toward those that have increased importance, because they indicate organisms having a greater impact on the host, or vice versa. Assemblages with similar associations might also be used to identify different taxa with similar functions as well as direct decisions concerning which of the myriad of unidentified variables should be examined further. This latter feature addresses the nature of data generated in this “omics era,” where most of the variables cannot be identified by simple database searches, but instead require procedures consuming considerable amounts of time and effort. Lastly, dissimilar relationships could provide key information, for example, in identifying relationships between host defense molecules and the bacteria they target.

## Conclusion

Our proposed method showed a significant utility and power in handling a data matrix of scatter plots. More importantly, this clustering procedure can be easily extended to the high dimensional case when one or more sets of variables needs to be analyzed. Moreover, the novel painting metrics we proposed can be easily extended to multidimensional clusters of multivariate plots.

Co-clustering is desirable over traditional one-dimensional clustering as it is more informative and easily interpretable while preserving most of the information contained in the original data; and it allows dimension reduction along both axes simultaneously and hence leads to a much more compact representation of the original data for subsequent analysis. Hence, our future study is to develop a new co-clustering method to deal with a data matrix of scatter plots.

Finally, although these methods were developed to analyze microbe-host interactions, we anticipate that this general approach will have utility for a wide range of investigations, including those examining relationships among gene expression profiles, metabolites, genes and epigenetic parameters.

## Appendix

### Data depth

Let *F* be a probability distribution in R^p^ with *P≥1* and *x⃗* a point in R^p^. The data depth at with respect to *F* is denoted by *D*(*F;x⃗*), which measures how deep (or central) the point *x⃗* is with respect to *F*. The larger, the deeper (or more central) the point with respect to F. Some commonly used data depth functions are listed as follows.

1. Mahalanobis Depth [19]:
  $$M_{h}D(F;\vec{x})=1/[1+{\left(\vec{x}-{\vec{\mu }}_{F}\right)}^{\prime }\sum_{F}^{-1}\left(\vec{x}-{\vec{\mu }}_{F}\right)],$$
  where *μ⃗_F_* and Σ*_F_* are the mean and variance-covariance matrix of *F*, respectively. The sample version of *M_h_D*(*F;x⃗*) is obtained by replacing *μ⃗_F_* and Σ*_F_* with their sample estimates.
2. Tukey Depth/Halfspace Depth [20]:
  $$TD(F;\vec{x})=\underset{Ç}{\inf}\{P(Ç):Ç\;\text{is}\;\text{a}\;\text{closed}\;\text{half}\;\text{space}\;\text{in}\;{\mathbb{R}}^{p}\text{containing}\;\vec{x}\}.$$
  The sample version of *TD*(*F;x⃗*) is *TD*(*F_n_;x⃗*) where *F_n_* is the empirical distribution.
3. Simplicial Depth [21]:
  $$SD(F;\vec{x})=P_{F}(\vec{x}\;\text{is}\;\text{inside}\;\text{the}\;\text{closed}\;\text{simplex}\;\text{whose}\;\text{vertices}\;\text{are}\;\{{\vec{X}}_{1},\cdots ,{\vec{X}}_{p+1}\}),$$
  where {*X⃗*_1_*,* ···, *X⃗_p_*_+1_} is a random sample from *F*. The sample version of *SD*(*F;x⃗*) is the fraction of the sample random simplexes containing the point *x⃗*.

### Q(F,G) vs. Q(G,F)

Consider two independent distributions *F* and *G*, and two variables X ~ F and Y ~ G. We present several examples to show the relationship between *Q(F,G)* and *Q(G,F)*. For simplicity, univariate normal distributions and Mahalanobis depth are adopted here.

Example 1: For $F=N(\mu_{0},\sigma_{0}^{2}),G=N(\mu_{0},\sigma_{1}^{2})$, and $\sigma_{1}^{2}>\sigma_{0}^{2}$, we have
  $$\begin{array}{l} Q(F,G)=P({\left(X-\mu_{0}\right)}^{2}/\sigma_{0}^{2}\geq {\left(Y-\mu_{0}\right)}^{2}/\sigma_{0}^{2})<1/2,\\ Q(G,F)=P({\left(Y-\mu_{0}\right)}^{2}/\sigma_{1}^{2}\geq {\left(X-\mu_{0}\right)}^{2}/\sigma_{1}^{2})<1/2, \end{array}$$
  and *Q(F,G)* + *Q(G,F)* = *1*,
Example 2: For $F=N(\mu_{0},\sigma_{0}^{2}),G=N(\mu_{0},\sigma_{1}^{2})$, and *μ_0_*≠*μ_1_*, we have
  $$\begin{array}{l} Q(F,G)=P({\left(X-\mu_{0}\right)}^{2}/\sigma_{0}^{2}\geq {\left(Y-\mu_{0}\right)}^{2}/\sigma_{0}^{2})<1/2,\\ Q(G,F)=P({\left(Y-\mu_{1}\right)}^{2}/\sigma_{0}^{2}\geq {\left(X-\mu_{1}\right)}^{2}/\sigma_{0}^{2})<1/2, \end{array}$$
  and *Q(F,G)* = *Q(G,F)*.
Example 3: For $F=N(\mu_{0},\sigma_{0}^{2}),\;G=N(\mu_{1},\sigma_{1}^{2})$, *μ_0_*≠*μ_1_*, and $\sigma_{1}^{2}>\sigma_{0}^{2}$, we have
  $$\begin{array}{l} Q(F,G)=P({\left(X-\mu_{0}\right)}^{2}/\sigma_{0}^{2}\geq {\left(Y-\mu_{0}\right)}^{2}/\sigma_{0}^{2})<1/2,\\ Q(G,F)=P({\left(Y-\mu_{1}\right)}^{2}/\sigma_{1}^{2}\geq {\left(X-\mu_{1}\right)}^{2}/\sigma_{1}^{2}), \end{array}$$
  and <1/2, =1/2, or >1/2.

## References

[1] Eisen MB, Spellman PT, Brown PO, Botstein D (1998). Cluster analysis and display of genome-wide expression patterns. Pro Natl Acad Sci.

[2] Gasch AP, Eisen MB (2002). Exploring the conditional coregulation of yeast gene expression through fuzzy k-means clustering. Genome Biol.

[3] Dhillon IS, Mallela S, Modha DS (2003). Information-theoretic co-clustering. KDD.

[4] Andreopoulos B, An A, Wang X, Schroeder M (2009). A roadmap of clustering algorithms: finding a match for a biomedical application. Briefings in Bioinfor.

[5] Jiang D, Tang C, Zhang A (2004). Cluster analysis for gene expression data: a survey. IEEE Trans Knowl Data Eng.

[6] Busygin S, Prokopyev O, Pardalos PM (2008). Biclustering in data mining. Computers & Operations Research.

[7] Madeira SC, Oliveira AL (2004). Biclustering algorithms for biological data analysis: a survey. IEEE/ACM Trans Comput Biol Bioinform.

[8] Van Mechelen I, Bock HH, De Boeck P (2004). Two-mode clustering methods: a structured overview. Stat Methods Med Res.

[9] Prelić A, Bleuler S, Zimmermann P, Wille A, Bühlmann P (2006). A systematic comparison and evaluation of biclustering methods for gene expression data. Bioinformatics.

[10] Liu RY, Singh K (1993). A quality index based on data depth and multivariate rank tests. J Am Stat Assoc.

[11] Zuo Y, He X (2006). On the limiting distributions of multivariate depth-based rank sum statistics and related test. Ann Stat.

[12] Li X, LeBlanc L, Elashoff D, Borneman J, Goodglick L (2010). Detecting Disease-Related Biological Neighborhoods by Human Mucosal Interface Metaproteome Analysis.

[13] Ahrenstedt O, Knutson L, Nilsson B, Nilsson-Ekdahl K, Odlind B (1990). Enhanced local production of complement components in the small intestines of patients with Crohn’s disease. N Engl j Med.

[14] Broedl UC, Schachinger V, Lingenhel A, Lehrke M, Stark R (2007). Apolipoprotein A-IV is an independent predictor of disease activity in patients with inflammatory bowel disease. Inflamm Bowel Dis.

[15] Foell D, Kucharzik T, Kraft M, Vogl T, Sorg C (2003). Neutrophil derived human S100A12 (EN-RAGE) is strongly expressed during chronic active inflammatory bowel disease. Gut.

[16] Greenstein AJ, Sachar DB, Panday AK, Dikman SH, Meyers S (1992). Amyloidosis and inflammatory bowel disease. A 50-year experience with 25 patients. Medicine (Baltimore).

[17] Hansen JJ, Holt L, Sartor RB (2009). Gene expression patterns in experimental colitis in IL-10-deficient mice,” Inflamm. Bowel Dis.

[18] Larsson AE, Melgar S, Rehnström E, Michaëlsson E, Svensson L (2006). Magnetic resonance imaging of experimental mouse colitis and association with inflammatory activity. Inflamm Bowel Dis.

[19] Mahalanobis PC (1936). On the generalized distance in statistics. Proceedings of the National Academy of India.

[20] Tukey JW (1974). Mathematics and picturing data. Proceedings of the International Congress of Mathematicians, Vancouver.

[21] Liu RY (1990). On a notion of data depth based on random simplices. Ann Stat.

